# Patients’ and clinicians’ perspectives on relevant treatment outcomes in depression: qualitative study

**DOI:** 10.1192/bjo.2020.27

**Published:** 2020-05-04

**Authors:** Kaying Kan, Frederike Jörg, Erik Buskens, Robert A. Schoevers, Manna A. Alma

**Affiliations:** University of Groningen, University Medical Center Groningen, University Center for Psychiatry, Rob Giel Research Center, Interdisciplinary Centre for Psychopathology and Emotion Regulation, the Netherlands; GGZ Friesland, Research Department, Leeuwarden; and University of Groningen, University Medical Center Groningen, University Center for Psychiatry, Rob Giel Research Center, Interdisciplinary Centre for Psychopathology and Emotion Regulation, the Netherlands; University Medical Center Groningen, Department of Epidemiology, Groningen; and University of Groningen, Faculty of Economics and Business, the Netherlands; University of Groningen, University Medical Center Groningen, University Center for Psychiatry, Interdisciplinary Centre for Psychopathology and Emotion Regulation, the Netherlands; University of Groningen, University Medical Center Groningen, Department of Health Sciences, Applied Health Research, Groningen, the Netherlands

**Keywords:** Depressive disorders, value-based healthcare, PROMS

## Abstract

**Background:**

Although symptomatic remission is considered the optimal outcome in depression, this is not always achieved. Furthermore, symptom indicators do not fully capture patients’ and clinicians’ perspectives on remission. Broader indicators of (partial) remission from depression should be considered.

**Aims:**

To investigate relevant outcomes of depression treatment in specialist care from patients’ and clinicians’ perspectives and to investigate whether these perspectives differ from each other.

**Method:**

Three focus groups with 11 patients with depression and seven semi-structured interviews with clinicians were conducted exploring their perspectives on remission. All interviews were audio-recorded and transcribed verbatim. We analysed the transcripts thematically using the phenomenologist approach.

**Results:**

Independently, both patients and clinicians perceived the following outcomes relevant: restoring social functioning and interpersonal relations, regaining quality of life and achieving personal goals. All clinicians emphasised symptom reduction and satisfaction with treatment as relevant outcomes, whereas the former was not an obvious theme in patients. Unlike clinicians, patients made a clear distinction between treatment outcomes in first versus recurrent/chronic depression.

**Conclusions:**

Classically defined study outcomes based on symptom resolution only partly reflect issues considered important by patients and clinicians in specialist depression treatment. Incorporating patients’ and clinicians’ perspectives in the development of measurable end-points makes them more suitable for use in trials and subsequent translation to clinical practice. Furthermore, evaluating patients’ perspectives on treatment outcomes helps in the development of tailored interventions according to patients’ needs.

Evaluation of depression treatment effectiveness may be less straightforward compared with other medical conditions. Multiple definitions are used, for example remission, response or recovery from depression, and inconsistencies within definitions exist.^1–3^

Remission is widely recognised as the most favourable outcome of treatment for depression and primarily relies on changes in the amount and severity of depressive symptoms.^4,5^ In clinical trials, depressive symptoms are measured by clinician-rated or patient-rated depression rating scales, which is a pragmatic way to determine treatment effects. Although the use of symptom rating scales is important for objectively measuring treatment outcomes, this might not be optimal in the case of depression. First, clinical trials often use treatment outcomes defined by researchers and the clinical community,^6,7^ instead of reflecting patients’ values regarding relevant treatment outcomes.^8^ Second, depression is a mental disorder with a high probability of recurrence and chronicity.^9^ In specialist mental healthcare, around 85% of patients with major depressive disorder experience a recurrence within 15 years.^10^ In those patients, remission might not be a realistic treatment outcome but would rather be an exception. Indeed, many remitted patients continue to experience ongoing deficits in functioning or quality of life.^11,12^

Symptom-based scales will likely remain the standard for treatment outcome assessment in randomised controlled trials and clinical practice. However, it may be worthwhile to develop measures that assesses relevant domains other than symptom resolution.^13^ Incorporating patients’ perspectives and attitudes towards health and illness, and taking a more patient-centred approach in the assessment of treatment outcomes has gained greater interest recently.

## Relevant outcomes of treatment

Patients, their spouses and the community demand treatment that produces relevant outcomes, i.e. significant return on investment.^14,15^ Patients that actively engage in treatment decisions have higher satisfaction scores and better clinical outcomes.^16,17^ Assessment of treatment effects may be biased if it is based on treatment outcomes that have limited relevance to patients and clinicians in the consulting room, having a negative impact on policy decision-making.

Little evidence is available about what patients in specialist mental healthcare who have experienced depression would define as relevant outcomes of treatment. A study performed in primary care demonstrated that patients value a broad range of indicators of recovery from depression, for instance, managing the depression, functioning as before and enjoying activities as before the depression.^18^ In a quantitative study, patients perceived symptom resolution as only one important factor. The presence of features of positive health (for example optimism), a return to one's usual level of functioning, and feeling like your usual self, were, according to patients, better indicators of remission from depression.^5^ Finally, two studies investigating relevant treatment goals showed that improvements in functioning in social and occupational domains were also considered important.^19,20^

To be able to develop treatments that are in line with patients’ needs and to provide effective specialist depression care, it is crucial to know what patients value as relevant outcomes of depression treatment in specialist depression care. In addition, it is important to know what clinicians perceive as relevant outcomes of treatment for their patients. Discrepant views may well bring out diverging expectations, leading to disappointment and treatment failure, unnecessary prolongation of treatment or treatment discontinuation. To our knowledge, clinicians’ perspectives on relevant outcomes of depression treatment have not been explicitly studied before. The aims of the study are (a) to investigate patient-relevant outcomes and clinician-relevant outcomes of depression treatment, and (b) to investigate whether any discrepancies exist between patients’ and clinicians’ views regarding these relevant outcomes.

## Method

Data for this qualitative study were collected by means of focus group interviews to stimulate exchange of views and experiences between patients. Due to their time constraints, we conducted semi-structured interviews with clinicians.

### Ethical aspects

In line with the Dutch Medical Research involving Human Subjects Act, the Medical Ethics Review Board (METc) of the University Medical Center Groningen exempted this research from full review. A waiver from the METc was obtained because there was not an infringement of the physical and/or psychological integrity of the participants. Prior to the start of the focus groups, written informed consent was obtained from all participants. All participants agreed on audiotaping the interviews and usage for scientific research after anonymisation.

### Selection of participants

Both patients and clinicians in the specialist mental healthcare setting were selected by purposive sampling. Patients were eligible for participation in the study if they (a) have (or had) a depressive disorder as main diagnosis; (b) had experience with depression treatment, and (c) were willing to share their views and experiences on relevant treatment outcomes for depression treatment in a focus group.

Clinicians were eligible for participation in the study if they (a) treat(ed) patients with depressive disorders, and (b) were willing to share their views on relevant treatment outcomes for depression treatment in a semi-structured interview.

Recruitment of participants took place in the mental healthcare organisations connected to the Rob Giel Research center (RGOc; a collaboration of six regional mental healthcare providers in the Northern-Netherlands). Patient Councils of these mental healthcare organisations distributed flyers in waiting rooms and day care areas. The call was also posted on the RGOc website (www.rgoc.nl) and distributed via the RGOc newsletter. Clinicians were recruited via the RGOc network, RGOc website and RGOc newsletter. Interested participants could sign-up by sending an email or by telephone. One of the researchers (K.K.) approached all participants by telephone for an eligibility check and asked them via snowball sampling for the recruitment of other potential participants.

In total 18 individuals were eligible: 12 patients and 7 clinicians. One eligible patient dropped out of the study as he forgot to attend the focus group meeting.

### Data collection

We conducted four focus group interviews in three focus groups between November 2016 and June 2017. During the first interview, which was a pilot, we were unable to discuss all the topics of the interview guide. Therefore, these participants were interviewed a second time to discuss the undiscussed topics of the interview guide and to verify the results of the first interview.

The focus group interviews with patients were moderated by F.J. (PhD, psychologist and epidemiologist) and K.K. (MSc, health scientist). Both researchers are women and have previous experience and training in conducting and executing qualitative research. The researchers did not know the participants in advance nor did they have a therapeutic relationship with the patients. Therefore, a short introduction round took place prior to the start of the focus group interviews. The researchers went openly into the focus group interviews.

The interview guide for the focus group interview was pilot tested with the first focus group after which we made small adaptations. Topics in the interview guide included: personal experiences with treatment for depression and treatment goals, used outcomes during treatment, views on the definition of remission/treatment success for treatment evaluation.

The focus group interviews with patients took place in a meeting room in a mental healthcare organisation that was closest to where the patients lived. Besides participants and researchers, no one else was present during the focus group interviews. The number of participants in the focus groups varied between three and five.

Seven semi-structured interviews with clinicians were conducted by K.K. in May and June 2017. The interview guide for the semi-structured interviews included the same topics as the interview guide used for patients. The semi-structured interviews with clinicians took place in the consulting room of the practising clinician.

During the (focus group) interviews, we made field notes and analysed those as preparation for the next interview. The duration of the focus group interviews and semi-structured interviews ranged between 73 and 86 min and between 40 and 85 min, respectively. We continued with data collection until data saturation was reached.

### Data analysis

All focus group interviews and semi-structured interviews were audiotaped and transcribed verbatim. We made a summary of all interviews and returned the summaries to the participants for a member check. We used the phenomenology approach to examine participants’ subjective experiences on a particular phenomenon. Data were coded using thematic content analysis. Two interviews were open coded by two researchers (K.K. and F.J.) with paper and pencil for comparison. After comparison and agreement on the codes, all transcripts were coded using the software package ATLAS-ti version 8.0.40.0 (ATLAS.ti Scientific Software Development GmbH) to ensure systematic coding of the data. Subsequently, main themes were derived from the data and identified from the codes. Finally, we integrated main themes and research data to compare patients’ perspectives and clinicians’ perspectives using the one-sheet-of-paper approach.^21^ Quotations from the participants were translated from Dutch by a native English speaker and are presented to illustrate the themes and findings. See supplementary material (available online at https://doi.org/10.1192/bjo.2020.27) for the consolidated criteria for reporting qualitative research checklist that was used to report this research.

To ensure validity of the study data, peer debriefing took place on a frequent basis with a third researcher, M.A.A. (PhD, social scientist). From the start of the study (prior to data collection), during data collection, coding of the data and reporting of the findings, this unbiased researcher provided feedback to ensure credibility.

## Results

Table 1 presents the characteristics of the study sample. Out of 11 patients, 8 patients were women. The patients ranged in age from 22 to 69 years. Nine patients were currently in treatment for their depression. Two patients were currently not in treatment and were in remission and/or had finished treatment at the time of the focus group interview. Most patients experienced multiple depressive episodes.

**Table 1:** Characteristics of interviewed patients and clinicians

	Patients	Clinicians
*n*	11	7
Mean age, years (s.d.)	43.9 (14.6)	48.4 (13.6)
Male gender, *n*	3	1
Highest attained educational level, *n*		
High school	3	–
Vocational education	1	–
Bachelor degree	6	–
Master degree	1	–
Household status, *n*		
Single, no child(ren)	4	–
Singe, with child(ren)	1	–
Cohabiting/married, no child(ren)	2	–
Cohabiting/married, with child(ren)	4	–
Depression as main diagnosis, *n*	11	–
Currently in treatment, *n*	9	–
Position, *n*		
Specialised psychiatric nurse	–	1
Psychologist	–	2
Psychiatrist	–	4
Work experience, years: mean (s.d.)	–	16.6 (14.3)

Six clinicians were women and one was a man. They ranged in age from 32 to 64 years. The clinicians worked in five different mental healthcare organisations. We interviewed four psychiatrists, one specialised psychiatric nurse and two psychologists.

### Patients’ perspectives on relevant treatment outcomes in depression treatment

Main themes that emerged from the analysis of the data, relating to relevant treatment outcomes from the patient's perspective, are described below and examples of quotes for each theme are illustrated in Table 2.

**Table 2:** Quotes for each theme from the patient's perspective

Themes	Quotations for illustration
Social functioning and interpersonal relationships	Quote 1: ‘So the client's own picture of themselves [how the client themselves feels that they function], but also how those around them feel that they function. Because I think that's what's most important, if you can function more or less normally, like you used to.’ (Participant 12, man, age 52) Quote 2: ‘I was finally functioning without medication, and I thought that was fine. It is fine until another bump comes along and then you start all over again. If I ask myself now; I just want to be able to function again and, if necessary, with medication, like I did a few years ago. For me, that's my recovery.’ (Participant 3, man, age 52)
Prevention of future recurrences	Quote 3: ‘If you've been given um, enough things to hold on to to pull yourself up at times when you are sinking. Learning to recognize and know what you have to do about it. Identifying and tackling it.’ (Participant 17, woman, age 25). Quote 4: ‘Another way of dealing with it …, is to be able to relate success to your ability to deal with a setback yourself. Without having to go straight back into treatment or taking more pills, that when there are setbacks, a hard day, which in the past would have sent you straight into the abyss, now you have learned, first I have to do this and then I have to do that and watch out for this and so on…’ (Participant 1, man, age 60)
Acceptance of illness and managing the depression	Quote 5: ‘During my first depressive episode, I really wanted things to be just like they were before. Although I did think that that would never happen, it was in fact my one sole wish. And, um, well, it's turned out be very different now from before, but better actually. But it was, it's been quite a process to accept things and to make adjustments.’ (Participant 13, woman, age 41) Quote 6: ‘I see recovery as learning to deal with your situation and to keep going. Because it will never make me better. And that has determined, and still determines, how I live my life and how I deal with my disabilities, what I do and what I don’t do. Those are two aspects that the… um, come back every day. What do I do and what do I forget about? That’s what, that's what it actually boils down to.’ (Participant 16, woman, age 69).
Personal goals and societal expectations	Quote 7: ‘That you go shopping, go to work and have a social life, and that this can be too much for people, or whether your goals is in fact that you can at least have a social life again, or just go to work, that can differ from one client to the next. But the outside world says, you're not really part of things again unless you're working, and that's what I'd really like to do.’ (Participant 3, man, age 52). Quote 8: ‘There is, for example, another goal that I have: in my contact with others I want to be less troubled by certain things, but that's not the same as not having any symptoms any more. And in my view, a practitioner often tends to look from that perspective, if things are x and y, then z is automatically the case, whereas it isn't always like that. Sometimes I can feel really good.’ (Participant 2, woman, age 22). Quote 9: ‘For almost everyone I can think of an example, with all the questionnaires [routine outcome monitoring questionnaires/symptom rating scales] that you have to fill in, that at some time they say, oh, you're doing a lot better, and that you definitely don't feel that yourself. So um, that's not the whole story.’ (Participant 1, man, age 60)

#### Social functioning and interpersonal relationships

The majority of patients mentioned goals related to social functioning (defined as an individual's ability to perform and fulfil normal social roles^22^) and interpersonal relationships as important goals of depression treatment. Normalisation of social functioning was considered important (Table 2, quote 1). It included getting out of bed, continuing normal daily activities and functioning as before the depression. One patient stated that it was acceptable to use antidepressant medication, if necessary, for obtaining normalisation of social functioning (Table 2, quote 2). Patients saw undertaking activities again with friends and family as a good indicator of social functioning.

However, patients who had experienced multiple depressive episodes or patients who were diagnosed with chronic depression had a different view on functioning. They stressed that they needed to find new ways of functioning they would consider as satisfactory given circumstances, even though it would not quite be in the same way as before, as illustrated in the next quote:

‘You can also find other ways, can't you? Functioning very differently from how you used to, and yet, um, find satisfaction. That you've found a new mode, let's say. So you could still have symptoms, but you have improved as it were.’ (Participant 13, woman, age 41)

Over one-third of the patients mentioned that their depression severity and stage of life determined their treatment goals related to functioning: young patients strived for returning to a full life, such as being part of the labour force, starting a family, having life goals like any other person, whereas middle-aged patients adjusted their goals and ambitions. They focused especially on personal relationships and functioning well within their family again. Being ‘free of depressive symptoms’ was not mentioned by any patient as a relevant treatment goal when patients experienced recurrent depression or became chronically depressed.

#### Prevention of future recurrences

More than half of the patients mentioned that they perceived long-term outcomes of depression treatment as very meaningful. They considered it important to learn how to integrate techniques on how to cope with depression in daily life and to obtain skills for the prevention or early signalling of future depressive episodes (Table 2, quote 3 and 4).

#### Acceptance of illness and managing the depression

As a result of patients experiencing several depressive episodes and remaining vulnerable, it appeared that accepting that depression is part of a patient's life was a way forward. One could still have a good life, and managing the depression became a goal in half of the interviewed patients as illustrated in the following quote and in Table 2 (quote 5 and 6).

‘…At some point you start to adjust your expectations and at some point you also realize okay well, in three years’ time I'd like to be here and here, what do I have to do to achieve that? And that's what I've more or less achieved and now I also think, okay and I'll never be entirely rid of it and I'll continue to have that vulnerability.’ (Participant 2, woman, age 22)

#### Achievement of personal goals and societal expectations

The majority of patients had personal goals that were set during their depression treatment, for example improving self-esteem, absence of suicidal thoughts, not being a burden to others, or structure in daily routine. One-third of the patients mentioned that their own expectations and goals changed after experiencing several depressive episodes, realising that full recovery is not attainable.

Some patients experienced societal pressure during treatment. They felt that their treatment goals were not always in line with those set by the treating clinician, mental healthcare organisation or society, as illustrated by quote 7 and 8 in Table 2. Finally, some patients got the impression that clinicians focused too much on having no residual symptoms as the clinical end-point, and that clinicians relied too much on depression severity scales, without looking at the individual patient (Table 2, quote 9).

### Clinicians’ perspectives on relevant treatment outcomes in depression treatment

Main themes identified from the clinician's perspective are described below and illustrated in quotes for each theme in Table 3.

**Table 3:** Quotes for each themes from the clinician's perspective

Themes	Quotations for illustration
Symptom reduction/clinical improvement	Quote 1: ‘The aim in fact is to always have an improvement in the symptoms. Usually measured by means of a questionnaire but also from a clinical point of view and what the patient tells you.’ (Participant 6, woman, age 37) Quote 2: ‘There are of course a number of symptoms that you look at. Um, both in mood and in activity, and in sleeping and eating and restoring contacts and, um, a reduction in anxiety. Um, so in fact you include all symptoms. You can do this using all sorts of questionnaires [measurement scales]. But this often brings you to a medical history and consultation, which can provide a lot of information.’ (Participant 8, woman, age 53)
Social functioning and interpersonal relationships	Quote 3: ‘Of course, you watch out for things: has someone become more active? Have they taken up their roles, their social and personal roles again? That's what we focus on in practice. So it's not just clinical, but, um, simply, “I notice I can take my child to school again.” “I'm getting up at 8 o'clock again.” “I have started running again.”’ (Participant 4, woman, age 60). Quote 4: ‘And as to whether or not you should go back to work, well, I'm not society, but I think it would very good if someone has a network again, that they have some form of social contact that goes a little further than the cashier at the grocery store, that someone builds up a network again, that people don't just think …, they take their pills and the worst is over, no, you also have to ensure that that things continue to go well, because it's an illness that's very chronic. And that's the biggest danger, that you're satisfied too soon. And that you therefore have to say to people, you have keep going or something like that, but that you have to consider that every time.’ (Participant 10, woman, age 59)
Patient satisfaction and quality of life	Quote 5: ‘Yeah, through a reduction in symptoms. And of course you also still have quality of life.’ (Participant 4, woman, age 60) Quote 6: ‘That you take as a guide: are you happy about it and have you achieved your goals?’ (Participant 9, woman, age 32) Quote 7: ‘At a certain point during treatment you simply notice that someone, um, and that it's actually been stable for quite some time. That the remission, um, you've had the remission for several weeks. That they themselves also come across differently and say that things are going well.’ (Participant 7, woman, age 34)
Achievement of predetermined personal goals	Quote 8: ‘I look at the level of symptoms, but I also look at whether someone has achieved their goals, um … So you really look at, what does someone want, what do they want, we try to make goals as specific as possible, in other words, what does someone want to change and to have achieved? And I then include that and we then do an evaluation. [the example cited is:] for example, I want to start exercising once a week again, it could be. Or someone says, I want more peace in my mind. Well, you have to make that more specific of course. Um, very often it involves picking up certain things again or doing less. Um, or fewer negative thoughts, thinking more positively about myself, those kind of things.’ (Participant 7, woman, age 34) Quote 9: ‘You draw up a treatment plan. And in the treatment plan, yeah, you indicate what you, what you in fact, what you want to achieve. Ideally, um, the objective should be achieved. Or in any case, um, should offer good prospects of being achieved.’ (Participant 5, man, age 64)

#### Symptom reduction/clinical improvement

All clinicians mentioned symptom reduction or clinical improvement as an important treatment outcome in practice. They focus specifically on the main symptoms of depression: low mood, loss of pleasure in activities and concentration. In addition, the majority of clinicians also use depression severity rating scales to evaluate symptom reduction or clinical improvement (Table 3, quote 1 and 2).

#### Social functioning and interpersonal relationships

All clinicians mentioned that patients should be able to take up former social and personal roles, as illustrated in the following example and quote 3 in Table 3.

‘That it gradually improves in terms of mood, that the level of activity improves, do they go back to school or not, do they start a course, do they leave the house, sometimes it's …, it depends how serious it is, to also look at that, not only to have the symptoms gone but also that they become active again and, say, resume their normal development somehow and keep going.’ (Participant 10, woman, age 59)

Participating at work was not seen as a necessary goal of treatment from the clinician's perspective (Table 3, quote 4).

#### Patient satisfaction and quality of life

The majority of clinicians noted that quality of life and patient satisfaction with treatment or satisfaction with functioning were also relevant when determining whether depression treatment was successful (Table 3, quote 5, 6 and 7). More than half of the clinicians mentioned that they ask whether their patients are satisfied with the results of treatment.

#### Achievement of predetermined personal goals

Most clinicians mentioned that achieving goals defined at the start of the treatment was a relevant indicator of treatment success (Table 3, quote 8). According to clinicians, these predetermined goals mainly relate to personal goals and may differ between patients, but should preferable be agreed upon by patient and clinician. One clinician mentioned that it would be helpful to base treatment success on achievement of predetermined treatment goals, as it is hard to tell whether a patient is in full remission (Table 3, quote 9).

### Similarities and discrepancies between patients’ and clinicians’ perspectives

In Table 4 relevant outcomes according to both perspectives are summarised. Restoring social functioning and interpersonal relations, regaining quality of life, and achieving personal goals were mentioned most often as important indicators of treatment success by both clinicians and patients. In the last column of the table, we have added outcomes that are frequently used in randomised controlled trials for comparison with the outcomes in our study.^1,2^ Apparently, in trials reduction of symptoms, i.e. (time to) recovery, remission, response, or a functional/administrative outcome are considered relevant outcomes.

**Table 4:** Discrepancies and similarities from patients’ perspective, clinicians’ perspective and outcomes used in randomised controlled studies

Outcome measure	Patient-relevant outcomes	Clinician-relevant outcomes	Most used outcomes in randomised controlled trials
Symptom reduction/clinical improvement	✓^a^	✓	✓
Social functioning	✓	–	–
Managing the depression	✓	–	–
Acceptance of the depression	✓	–	–
Absence of future recurrences/obtain skills for relapse prevention	✓	–	–
Achievement of personal goals	✓	✓	–
Patient satisfaction with treatment	–	✓	–
Maintenance quality of life	✓	✓	–
Statistical tests	–	–	✓
Time to recovery	–	–	✓
Cut-off score on depression rating scale (remission)	–	✓^b^	✓
% change on depression rating scale (response/recovery)	–	✓^b^	✓
Functional criteria (for example hospital discharge or admission, change in assigned treatment)	–	–	✓

✓, present.

a. ‘Functioning as before’.

b. Results depression rating scales + clinical judgement.

Importantly, evident discrepancies between clinicians’ judgements and patients’ perspectives also emerged. Clinicians emphasised symptom reduction as a relevant treatment outcome, whereas for patients this was not a prominent theme. Although normalisation of social functioning and interpersonal relationships may go hand in hand with symptom reduction, some patients argued clinicians focused too much on being free of symptoms, whereas patients would rather learn how to manage their depression. Next, only patients and not clinicians made a clear distinction between treatment outcomes in a first-episode of depression versus recurrent/chronic depression. Notably, clinicians mentioned patient satisfaction with treatment as a relevant outcome in the treatment of depression.

## Discussion

### Main findings and comparison with findings from other studies

This qualitative study provides new insights into what patients and clinicians perceive as relevant outcomes in the treatment of depression in the specialist care setting. From a patient's perspective, the main themes identified were social functioning and interpersonal relationships, prevention of future recurrences, managing depression and achieving personal goals. Clinicians on the other hand were mainly focused on symptom reduction and clinical improvement, social functioning and interpersonal relationships, patient satisfaction and achievement of predetermined personal goals. Patients and clinicians agreed on the majority of issues, but differences were also found.

Importantly, usual end-points defined in trials, i.e. remission and response, only to a limited extent appear to reflect what matters in clinical practice. This may indicate that the efficacy observed in trials may not reflect actual preferences, which in turn results in disappointment with treatment effects. The focus group interviews undeniably demonstrated that patients perceived other outcomes relevant in addition. Especially after experiencing several depressive episodes, patients adapt their treatment goals. Our results indicate that we may need to use different end-points when treating a first episode versus recurrent or chronic depression, so that treatment goals and success can reflect all stages of the disease.^23^ To inform more extensively on clinically relevant results, Rush et al proposed including a second end-point, such as daily functioning, in clinical trials in difficult-to-treat depression.^24^

As in other chronic diseases, patients have reported still experiencing a good quality of life despite the fact that they have not fully recovered or achieved remission, the so called ‘disability paradox’.^25^ Clinicians on the other hand, emphasised symptom reduction, treatment satisfaction and achievement of predetermined treatment goals, of which the latter are not generally taken into account in clinical trials either.

There is an ongoing debate whether the World Health Organization definition of health, described as ‘a state of complete physical, mental and social well-being and not merely the absence of disease or infirmity’^26^ still fits in a world where chronic diseases are highly prevalent. Newly proposed definitions suggest including the ability of people to develop strategies to cope with the disease while maintaining their perceived quality of life.^27^ Learning to cope with depression and learning to function satisfactorily are in line with the proposed definitions of health, especially in chronic conditions.

By using qualitative methods, patients and clinicians can elaborate freely on what they consider important without being guided by suggestions from researchers, pre-defined questionnaires or clinical expectations. Our findings are in accordance with findings from the few previous studies that were performed on defining recovery from depression, especially regarding functioning in several domains and returning to one's usual level of functioning.^5,18–20,28^ In addition, we noted that patients gradually modify their treatment goals after several reoccurrences, which, as far as we know, has not been described before. Interestingly, relevant outcomes related to different domains of functioning (such as physical, social) are also found to be important in other disorders (for example heart failure, diabetes, psychosis).^29–31^

### Limitations

In this study, a few factors should be taken into consideration. First, most of the interviewed patients had either chronical depression or had gone through several depressive episodes, which was a representation of the specialist mental healthcare treatment setting. In the Netherlands, specialist mental healthcare providers deliver in-patient, out-patient and community care treatment to patients with severe mental health problems. In contrast, patients with mild-to-moderate non-complicated psychiatric disorders receive treatment from a general practitioner with additional support from a specialised mental healthcare nurse. Alternatively, they may be referred to generalist mental healthcare. Patients with a single episode only were less represented. However, the patients in our study that experienced multiple episodes of depression mentioned the same relevant treatment outcomes for a first episode as patients who experienced a single episode only. Our study sample is likely to be representative for patients in our catchment area. The ratio of men to women diagnosed with depression is 2:3.

Second, most participants were of White ethnicity. Culture and ethnicity might have influenced treatment goals deemed relevant in depression care. Third, within the varied group of clinicians not all professions were equally well represented. However, we verified our results by grouping the type of clinicians and it appeared the results were very consistent between the professions. Therefore, we think that our sample of clinicians has provided representative themes, and data saturation was reached. Finally, a focus group interview with both patients and clinicians might have resulted in more in-depth discussions and interaction between both views. On the other hand, this could also have introduced social desirability bias in patients.

### Implications

The results of this study are relevant for both research and clinical practice. Traditional end-points used in clinical trials only partly reflect factors that are deemed relevant to patients and clinicians alike. Several outcomes considered relevant by patients and clinicians, such as functioning and quality of life, would be relatively easy to incorporate in outcome assessment for depression, as validated questionnaires are available. In addition, by developing or adding questionnaires related to the achievement of personal goals and patient's treatment satisfaction, this field would move even further towards value-based care. The current study therefore provides relevant input for the further development of patient-reported outcome measures. The development of such self-reported instruments necessitates direct input from patients, which is still broadly disregarded in conventional research.^15,32^

Likewise, goal-setting appears to be very important for treatment evaluation from both the patients’ and clinicians’ perspectives, but is barely used in randomised controlled trials to evaluate treatment. To ensure that patients and clinicians speak the same language, they should be more explicit in the common goals they have in mind for treatment. How patients and clinicians can improve common goal-setting warrants further investigation.

To conclude, in treatment-outcomes research, the focus should go beyond symptom resolution. Both patients and clinicians value other outcomes, especially in recurrent or chronic depression. When treatment efficacy and effectiveness are measured in terms of value-based end-points, treatment outcomes are more meaningful, and improvement in treatment success rates becomes achievable.

## Data Availability

As the participant consent for the collection of data did not explicitly or implicitly include details of sharing their anonymised data, we are legally and ethically not allowed to upload the data for sharing. Data are from the IMPROVE study, whose researcher may be contacted at k.kan@umcg.nl.

## Supplementary material

For supplementary material accompanying this paper visit http://dx.doi.org/10.1192/bjo.2020.27. click here to view supplementary material
